# Serum Metabolomic Profiling in Acute Alcoholic Hepatitis Identifies Multiple Dysregulated Pathways

**DOI:** 10.1371/journal.pone.0113860

**Published:** 2014-12-02

**Authors:** Vikrant Rachakonda, Charles Gabbert, Amit Raina, Lauren N. Bell, Sara Cooper, Shahid Malik, Jaideep Behari

**Affiliations:** 1 Department of Medicine, Divisions of Gastroenterology, Hepatology, and Nutrition, University of Pittsburgh, Pittsburgh, Pennsylvania, United States of America; 2 Metabolon, Inc., Durham, North Carolina, United States of America; 3 Hudson Alpha Institute for Biotechnology, Huntsville, Alabama, United States of America; Korea University, Republic of Korea

## Abstract

**Background and Objectives:**

While animal studies have implicated derangements of global energy homeostasis in the pathogenesis of acute alcoholic hepatitis (AAH), the relevance of these findings to the development of human AAH remains unclear. Using global, unbiased serum metabolomics analysis, we sought to characterize alterations in metabolic pathways associated with severe AAH and identify potential biomarkers for disease prognosis.

**Methods:**

This prospective, case-control study design included 25 patients with severe AAH and 25 ambulatory patients with alcoholic cirrhosis. Serum samples were collected within 24 hours of the index clinical encounter. Global, unbiased metabolomics profiling was performed. Patients were followed for 180 days after enrollment to determine survival.

**Results:**

Levels of 234 biochemicals were altered in subjects with severe AAH. Random-forest analysis, principal component analysis, and integrated hierarchical clustering methods demonstrated that metabolomics profiles separated the two cohorts with 100% accuracy. Severe AAH was associated with enhanced triglyceride lipolysis, impaired mitochondrial fatty acid beta oxidation, and upregulated omega oxidation. Low levels of multiple lysolipids and related metabolites suggested decreased plasma membrane remodeling in severe AAH. While most measured bile acids were increased in severe AAH, low deoxycholate and glycodeoxycholate levels indicated intestinal dysbiosis. Several changes in substrate utilization for energy homeostasis were identified in severe AAH, including increased glucose consumption by the pentose phosphate pathway, altered tricarboxylic acid (TCA) cycle activity, and enhanced peptide catabolism. Finally, altered levels of small molecules related to glutathione metabolism and antioxidant vitamin depletion were observed in patients with severe AAH. Univariable logistic regression revealed 15 metabolites associated with 180-day survival in severe AAH.

**Conclusion:**

Severe AAH is characterized by a distinct metabolic phenotype spanning multiple pathways. Metabolomics profiling revealed a panel of biomarkers for disease prognosis, and future studies are planned to validate these findings in larger cohorts of patients with severe AAH.

## Introduction

Acute alcoholic hepatitis (AAH) is a systemic condition that affects 10–35% of individuals with chronic ethanol use [1], [2]. AAH is characterized by the rapid development of jaundice, hepatomegaly, and features of the systemic inflammatory response syndrome (SIRS). Maddrey's Discriminant Function (mDF) is the most commonly used scoring system to assess severity of AAH [3], [4], and an mDF >32 with or without hepatic encephalopathy is associated with up to 39% six-month mortality [5].

Macrovesicular steatosis, or intrahepatic lipid accumulation, is a key histologic feature of AAH [6]. Early studies of ethanol-fed rodents have suggested that steatosis may be due to alterations in hepatic lipid metabolism, including enhanced de novo lipogenesis, reduced hepatic very low density lipoprotein (VLDL) secretion, and altered fatty acid oxidation (FAO) [7]–[11]. More recent work, however, has implicated extrahepatic derangements in global energy homeostasis such as dysregulated adipose tissue triglyceride hydrolysis and altered substrate utilization [12].

While animal models have yielded valuable insights into the pathogenesis of alcoholic steatosis, the relevance of these findings to the development of human alcoholic hepatitis remain less clear. Therefore, the aim of our study was to describe systemic metabolic phenotypes in acute alcoholic hepatitis. We performed an unbiased serum metabolomics analysis in a prospectively-collected cohort of patients hospitalized with severe AAH and in stable subjects with alcoholic cirrhosis to 1) characterize alterations in metabolic pathways associated with acute AAH, and 2) identify potential metabolic biomarkers for disease severity and prognosis.

## Materials and Methods

### Study Population

This prospective, case-control study was approved by the Institutional Review Board (IRB) at the University of Pittsburgh in 2010. Twenty-five adults hospitalized with severe AAH (i.e., mDF >32) were recruited at the University of Pittsburgh Medical Center in Pittsburgh, Pennsylvania from October 2010 to December 2012. Inclusion criteria included: total bilirubin level of ≥3 mg/dL, aspartate aminotransferase (AST): alanine aminotransferase (ALT) activity >2 with both levels <500 U/L, and the presence of either hepatomegaly or steatosis by ultrasound or computed tomography (CT) imaging in an individual with heavy, chronic ethanol use. Patients were excluded if other chronic liver diseases, including alpha-one antitrypsin deficiency, autoimmune hepatitis, hereditary hemochromatosis, Wilson's disease, drug-induced liver injury, primary biliary cirrhosis, primary sclerosing cholangitis or Budd-Chiari Syndrome were present. Patients with chronic viral hepatitis, however, were included. Individuals who last consumed ethanol more than 6 weeks prior to clinical presentation were excluded. Informed consent was obtained from either the subject or an authorized representative prior to participation. The clinical diagnosis of AAH was confirmed by two attending hepatologists (JB, SM) experienced in the treatment of alcoholic hepatitis.

As most patients with AAH exhibit histologic evidence of micronodular cirrhosis [13], [14], a control group of 25 clinically stable subjects with alcoholic cirrhosis was derived from the IRB-approved University of Pittsburgh Center for Liver Diseases Research Registry. All registry participants previously consented to inclusion in the registry and provided serum samples for research use. All patient records were reviewed to confirm the absence of acute alcoholic hepatitis.

### Clinical and Demographic Information

Clinical and demographic data (**Table 1**) were obtained at the time of enrollment. In patients with severe AAH, Maddrey's discriminant function (mDF) was calculated using the following formula [4]:

**Table 1:** Clinical and Demographic Characteristics of Patients.

Characteristics	AAH (N = 25)	Cirrhosis (N = 25)	*P*
**Age, yrs**	50 (45–56)	55 (46–57)	NS
**Male, n (%)**	17 (68%)	18 (72%)	NS
**Ethnicity, n (%)**			
**Caucasian**	23 (92%)	24 (96%)	NS
**Black**	2 (8%)	1 (4%)	NS
**Other Liver Disease, n (%)**	1 (4%)	11 (44%)	0.008
**Hepatitis C**	1 (4%)	10 (40%)	<0.001
**Hepatitis B**	0 (0%)	1 (4%)	NS
**Other**	0 (0%)	0 (0%)	NS
**BMI, kg/m^2^**	26.2 (23.4–33.0)	27.6 (25.3–30.1)	NS
**Diabetes, n (%)**	2 (8%)	3 (12%)	NS
**Discriminant Function**	52.5 (38.2–68.8)	N/A	N/A
**MELD**	26 (24–32)	11 (9–14)	<0.001
**Albumin, g/L**	2.0 (1.7–2.5)	3.2 (2.9–4.1)	<0.001
**Bilirubin, mg/dl**	15.0 (10.9–19.3)	1.3 (1.0–2.1)	<0.001
**AST, IU/L**	145 (100–235)	26 (20–51)	<0.001
**ALT, IU/L**	46 (38–64)	43 (32–63)	NS
**INR**	2.0 (1.8–2.5)	1.3 (1.2–1.5)	<0.001
**Creatinine, mg/dl**	1.0 (0.8–2.0)	0.8 (0.8–1.1)	NS
**WBC count, x10^3^/ml**	10.9 (7.5–14.5)	5.6 (4.2–6.8)	<0.001
**Hemoglobin, g/dl**	10.6 (10.0–11.8)	13.6 (12.7–14.9)	<0.001
**Platelet count, x 10^3^/ml**	127 (83–182)	89 (67–140)	NS
**30-day survival, n (%)**	18 (72%)	25 (100%)	<0.001
**90-day survival, n (%)**	16 (64%)	25 (100%)	<0.001
**180 day survival, n (%)**	14 (56%)	25 (100%)	<0.001

Baseline characteristics of patients. All values are expressed as medians and IQRs unless otherwise specified. Continuous variables were compared with Mann-Whitney *U* test. Categorical variables were compared with Fisher's exact test. N/A: not applicable; NS: not signifnicant (*p*>0.05).

MDF = [PT (sec) – control PT (sec)]×4.6+total bilirubin (mg/dl)

The laboratory control PT is 12.5 seconds at our institution. The Model for End Stage Liver Disease (MELD) score was determined from the following equation [15]:

MELD = (0.957×log [creatinine (mg/dl)]+0.378×log [total bilirubin (mg/dl)]+1.120×log (INR)+0.643)×10

Patients who underwent two or more dialysis treatments with one week of enrollment were assigned a creatinine value of 4.0 mg/dl when calculating MELD. Survival was determined using the Social Security Death Index.

### Metabolomics Analysis

Venous blood samples were collected from hospitalized patients with severe AAH within 24 hours of admission. Serum was obtained by centrifugation and stored at −80°C until testing. Global, unbiased metabolic profiling was performed as previously described [16].

### Data Acquisition and Statistical Analysis

Samples were analyzed over a two day period. Missing values for a given metabolite were replaced with the observed minimum detection value for that metabolite, based on the assumption that missing values were below the detection limit. Raw data counts for each sample were then normalized to the median value for each biochemical of interest.

Statistical analysis was performed using R (http://cran.r-project.org) and Stata version 11 (StataCorp, College Station, TX). Welch's *t* tests were carried out on log-transformed data to compare relative biochemical concentrations between experimental groups. False discovery rate (FDR) was estimated using *q* values to account for multiple comparisons.

Random forest (RF) analysis was performed on untransformed data as previously described to differentiate severe AAH and alcoholic cirrhosis cohorts [17]. Principal component analysis was utilized to determine variation between experimental groups [18], and integrated hierarchical clustering analysis was used to classify patient cohorts by groupings of metabolites [19]. To determine global patterns of metabolic pathway alterations, mass spectroscopic data were mapped in a targeted manner using the Metscape application within the Cytoscape platform [20], [21].

Demographic and clinical variables were presented as absolute frequencies, percentages, medians and interquartile ranges. The χ^2^ test was used to compare categorical variables between groups (or Fischer's exact test when expected values were ≤5), and the Mann Whitney *U* test was used for continuous variables. A two-tailed *p* value≤0.05 was considered statistically significant.

## Results

### Patient characteristics

Demographic and clinical features of the study population are presented in **Table 1**. Twenty-five patients were included in each cohort. Age, gender, and ethnicity were similar between groups. The prevalence of diabetes was similar, and both populations were overweight by mean BMI. More patients with alcoholic cirrhosis had viral hepatitis, but subjects with AAH exhibited increased MELD scores, AST levels and WBC counts with decreased hemoglobin and albumin. While all cirrhotic patients were alive after 6 months, only 56% of AAH patients survived beyond 180 days.

### Metabolomics Analysis: Overview and Classification of Patient Cohorts

A total of 420 distinct, named metabolites were identified in the serum samples. Changes in metabolite concentrations between cohorts were determined using the ratio of their group means, and these are displayed in **Table S1**. Overall, 234 statistically significant biochemical changes spanning multiple metabolic pathways were observed; 152 molecules were significantly increased in patients with severe AAH, while 82 were decreased. In addition, 17 biochemicals exhibited trends toward significant difference between groups (0.05<*p*<0.10).

**Figure 1:**
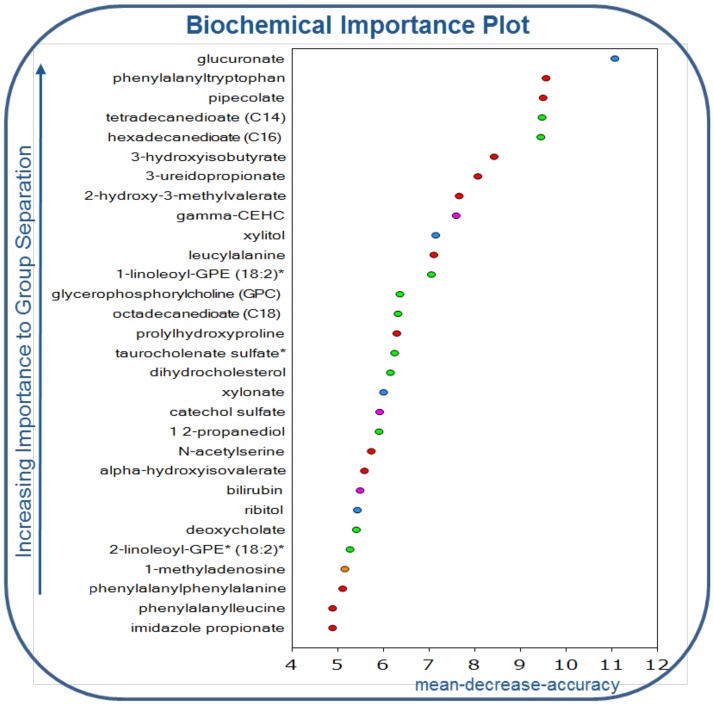
Random forest analysis of the thirty most important metabolites distinguishing patients with severe acute AAH from stable, alcoholic cirrhotic controls.

**Figure 2:**
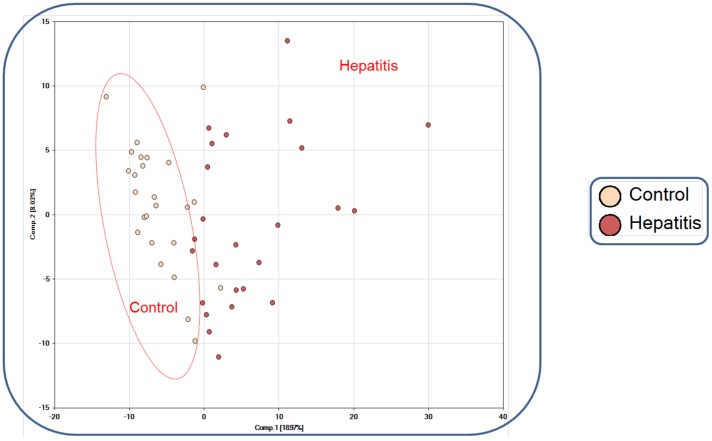
Principal component analysis (PCA) of metabolomics data reveals clustering of data into separate cohorts. Control: alcoholic cirrhosis; hepatitis: severe AAH.

Using RF analysis, we determined that the metabolomics data set classified samples into their respective groups with 100% accuracy. To assess the relative contribution of each variable, the mean decrease accuracy (MDA) metric was used [17], [22]. The 30 most important metabolites for the classification scheme are depicted in **Figure 1**. Next, principal component analysis (PCA) was carried out to show that the two groups were distinguishable by their metabolic signatures (**Figure 2**). Finally, unbiased integrated hierarchical clustering analysis demonstrated that differences in measured metabolites were grouped by subject cohorts (**Figure S1**). Together, PCA, RF analysis, and integrated hierarchical clustering methods demonstrate that AAH is characterized by distinct a metabolic profile.

**Figure 3:**
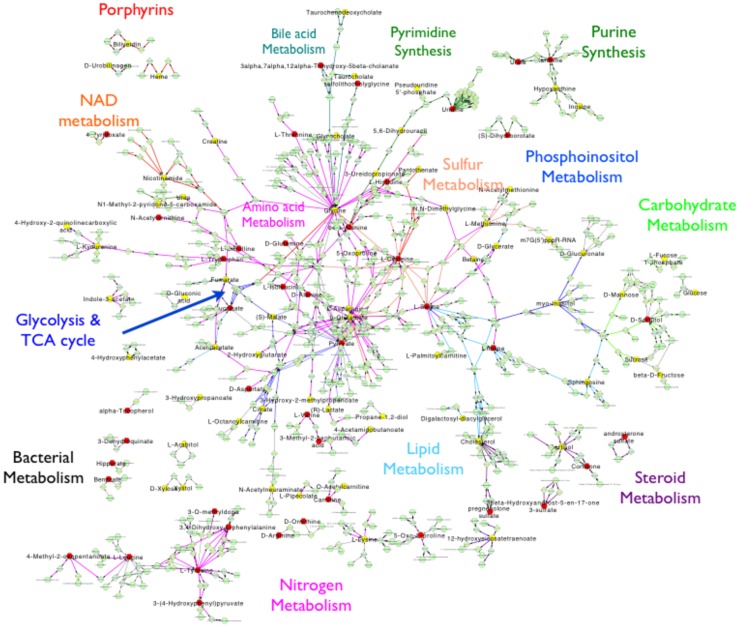
Global network analysis of relative measured metabolites in patients with alcoholic cirrhosis and severe AAH. Red nodes depict metabolites that are increased in severe AAH when compared to alcoholic cirrhosis; yellow nodes depict metabolites that are decreased in severe AAH when compared to alcoholic cirrhosis.

Targeted mapping of small molecules onto biochemical networks was performed to determine global patterns of metabolic alterations in AAH. Metabolic perturbations spanned multiple domains, including energy homeostasis pathways, bile acid handling, bacterial metabolism, nucleic acid turnover, antioxidant systems, and others (**Figure 3**).

### Lipid metabolism in severe AAH is characterized by enhanced adipose tissue lipolysis and altered fatty acid oxidation

Pathways related to lipid metabolism were significantly altered in severe AAH compared to alcoholic cirrhosis, and several biochemicals associated with lipid metabolism were identified in the RF analysis.

**Figure 4:**
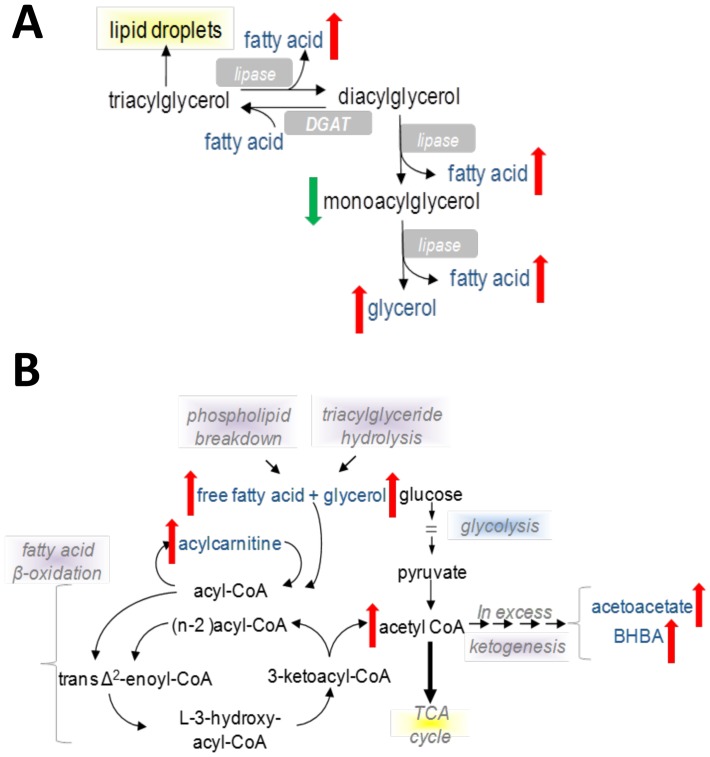
Schematic representations of altered triglyceride hydrolysis and mitochondrial fatty acid beta-oxidation in severe AAH. (A) Triglyceride hydrolysis. (B). Mitochondrial fatty acid beta-oxidation.

Elevations in several long chain free fatty acids (LCFAs), and in particular two essential fatty acids (eicosopentaenoate EPA; 20∶5n3, docosapentaenoate n3 DPA; 22∶5n6) were observed in patients with severe AAH (**Figure S2A**). This was accompanied by decreased monoacylglycerols with increased glycerol levels (**Figure S2B**). Overall these findings suggest that severe AAH is characterized by enhanced lipolysis (**Figure 4A**), and adipose tissue lipolysis may increase intrahepatic fatty acid influx in severe AAH. Potential etiologies for lipolysis upregulation include peripheral insulin resistance, enhanced sympathetic tone, or elevated stress hormone activity.

Beta oxidation is a primary fatty acid utilization pathway in the liver, and dysregulation of fatty acid beta oxidation has been described in both alcoholic and nonalcoholic liver disease [11], [23]. Beta oxidation primarily occurs in mitochondria; while short- (SCFA) and medium chain fatty acids (MCFA) can freely cross mitochondrial membranes, LCFA require carnitine shuttle systems to enter mitochondria. In patients with severe AAH, increased lipolysis was accompanied by metabolic changes suggestive of altered fatty acid beta oxidation. In the AAH cohort, significantly reduced levels of the SCFA valerate and also several MCFA were observed in conjunction with elevated acetylcarnitine and ketones (**Figure S3A, C**), indicating ongoing mitochondrial beta-oxidation. At the same time, multiple LCFA moieties and acylcarnitines were also elevated in the severe AAH cohort (**Figure S3B**), implying either 1) incomplete LCFA beta oxidation due to insufficient mitochondrial oxidative capacity that is overwhelmed by FA flux from adipose tissue or 2) defective carnitine shuttle activity (**Figure 4B** **)**.

**Figure 5:**
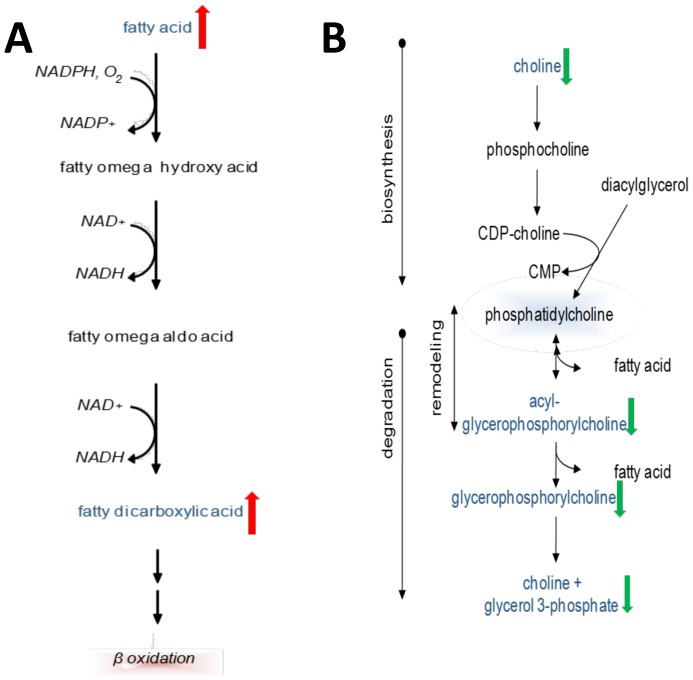
Schematic representations of altered fatty acid omega-oxidation and lysolipid and phospholipid turnover in severe AAH. (A) Fatty acid omega-oxidation. (B) Lysolipid and phospholipid turnover.

Omega oxidation is a minor pathway of fatty acid metabolism that occurs in peroxisomes and is primarily responsible for processing very long chain fatty acids (VLCFAs) into dicarboxylic acids. Omega oxidation is upregulated when beta oxidation pathways are insufficient for a given FA load [24]. In patients with severe AAH, serum levels of six dicarboxylic acids were significantly increased (**Figure S4,** **Figure 5A**), and three of these (tetradecanedioate, hexadecanedioate, octadecanedioate) were also identified in the RF analysis as important contributors for identification of severe AAH patients. These changes reflect enhanced omega oxidation in severe AAH and support the notion of inadequate beta-oxidative capacity for a FA disposal. Furthermore, dicarboxylic acids are known peroxisome proliferator-activated receptor α (PPARα) ligands and may regulate intrahepatic lipid partitioning in AAH [25].

### Deceased phospholipid and lysolipid turnover may reflect changes in plasma membrane remodeling in severe AAH

Phospholipids and lysolipids are the principal components of lipid bilayers in cell membranes. Phospholipids are composed of a diacylglycerol group, phosphate head group and an organic molecule, while lysolipids resemble phospholipids except for the replacement of diacylglycerol with monoacylglycerol. Phospho(lyso)lipid degradation and synthesis contribute to plasma membrane remodeling. Compared to cirrhotic patients, subjects with severe AAH exhibited significant reductions in three major phospholipid metabolites – choline, glycerophosphocholine (GPC) and glycerol-3-phosphate. Furthermore, the majority of detected lysolipids were decreased (**Figure 5B** **, Figure S5**). Two lysolipids – 1-linoleoylglycerophosphoethanolamine (1-linoleoyl-GPE) and 2- linoleoylglycerophosphoethanolamine (2-linoleoyl-GPE) – along with GPC were identified in the RF analysis as key metabolites for identification of severe AAH. Finally, increased levels of other important membrane components including cholesterol and sphingosine were observed (**Table S1**). Together, these findings may reflect significant reductions in cell membrane remodeling in patients with AAH.

### Alterations in bile acid composition and xenobiotic metabolism may indicate intestinal dysbiosis in severe AAH

**Figure 6:**
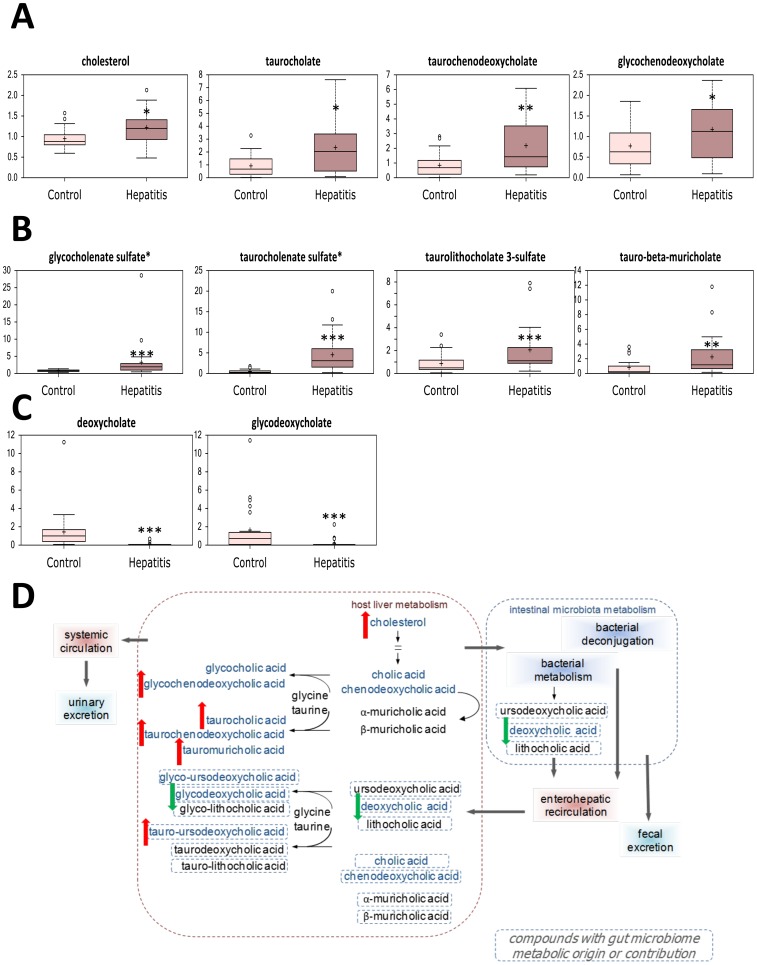
Alterations in serum bile acid levels in patients with severe acute alcoholic hepatitis (hepatitis) and stable alcoholic cirrhosis (control). (A) Secondary bile acids. (B) Sulfated bile acids. (C) Products of gut microbial bile acid metabolism. (D) Schematic representation of altered bile acid turnover in severe AAH. * *p*<0.05, ** *p*<0.01, *** *p*<0.001.

Cholestasis with associated elevation of serum bile acids is commonly encountered in alcoholic hepatitis, and accordingly, several secondary bile acids were increased in this cohort (**Figure 6A,D**). Sulfation is a key mechanism for increasing bile acid solubility for urinary excretion, and levels of three detected sulfated bile acid were elevated as well (**Figure 6B**). Both taurocholenate sulfate and deoxycholate were identified in the RF analysis, thus highlighting the importance of cholestasis as a key metabolic feature of severe AAH.

**Figure 7:**
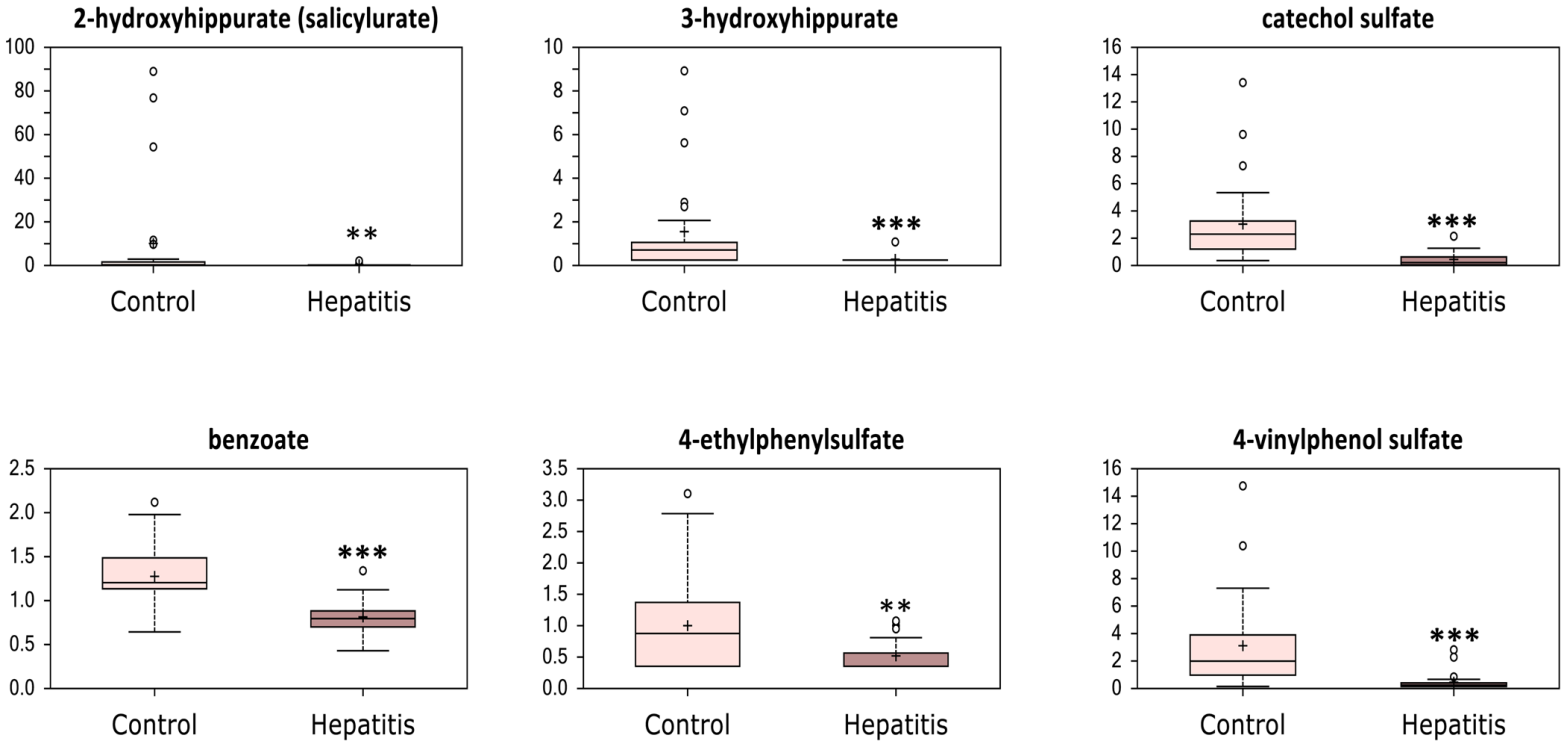
Alterations in serum levels of metabolites derived from intestinal microbial benzoate metabolism in patients with severe acute alcoholic hepatitis (hepatitis) and stable alcoholic cirrhosis (control). * *p*<0.05, ** *p*<0.01, *** *p*<0.001.

Conversely, the bile acids deoxycholate and glycodeoxycholate, were decreased (**Figure 6C**); as these bile acids are derived from intestinal bacterial metabolism, this may suggest altered gut microbial composition in severe AAH. Furthermore, several products of benzoate degradation were significantly reduced in patients with severe AAH, and this finding was corroborated by evidence of altered gut microbial benzoate metabolism (**Figure 7** **,** **Figure 3**). Together, these findings provide evidence of intestinal dysbiosis in patients with severe AAH.

### Severe AAH is characterized by changes in glucose utilization and tricarboxylic acid (TCA) cycle activity

**Figure 8:**
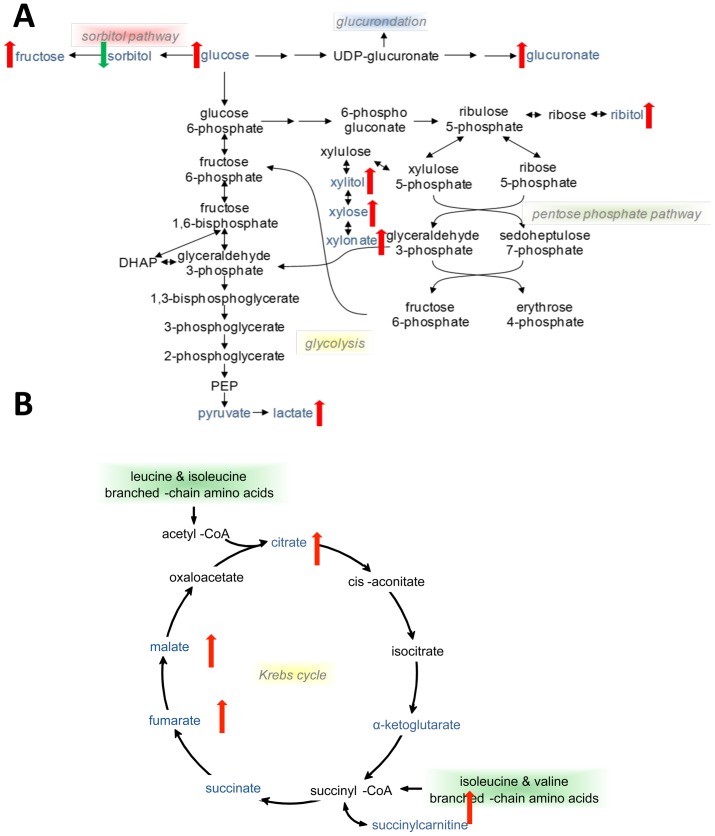
Schematic representations of altered serum metabolites involved in glucose utilization and TCA cycle activity in severe AAH. (A) Glucose utilization pathways. (B) TCA cycle.

In the absence of hepatic dysfunction or fasting, glucose is preferentially consumed to generate adenosine triphosphate (ATP) through glycolysis, the TCA cycle, and oxidative phosphorylation. In severe AAH, reduced glucose utilization was observed. Despite increased levels of serum lactate, elevations in glucose, fructose, and several sugar alcohols suggest glucose shunting away from glycolysis and towards the pentose phosphate pathway (PPP) (**Figure S6A,B**). In this context, increased lactate levels may reflect impaired hepatic or renal clearance. Furthermore, the PPP end product xylonate was identified in the RF analysis as a key marker of severe AAH. As the PPP is critical for generation of NADPH to neutralize reactive oxygen intermediates, elevations in PPP metabolites likely reflect increased oxidative stress in patients with AAH (**Figure 8A**).

Severe AAH was associated with a 15-fold increase in glucuronate. Additionally, glucuronate was identified as the most important molecule for AAH discrimination by RF analysis. As an important glucose derivative, glucuronate is conjugated to xenobiotics, bile acids, and bilirubin via UDP-glucuronosyl transferases (UGTs) to increase solubility and excretion. In particular, glucuronidation is essential for the elimination of bilirubin. In the AAH cohort, the median bilirubin level was significantly elevated at 15.0 mg/dl, suggesting that elevated glucuronate levels were related to either impaired or overwhelmed hepatic UGT capacity (**Figure 8A**).

Multiple intermediates of the TCA cycle, including malate, fumarate, succinate (in the form of succinylcarnitine) and citrate were increased in patients with severe AAH compared to cirrhotic controls (**Figure 8B** **, Figure S6D**); these findings are consistent with altered mitochondrial ATP production. Several potential TCA cycle substrates have been identified in this analysis, including products of FA metabolism and protein catabolism. For example, as there is evidence of ongoing FA beta-oxidation in severe AAH, acetylcarnitine end-products may drive Krebs cycle activity through increased acetyl-CoA availability. On the other hand, reduced glucose utilization via glycolysis may decrease pyruvate availability for conversion to acetyl-CoA.

### Changes in AA metabolite and peptide derivatives suggest increased protein catabolism for energy production

Amino acids provide yet another substrate for energy production, and marked changes in protein metabolism were noted in patients with severe AAH. In particular, significant reductions in all three branched-chain amino acids (BCAAs) were accompanied by increased levels of BCAA metabolites (**Figure S7A, B**). In addition, levels of TCA cycle intermediates derived from BCAA were increased in patients with severe AAH. As skeletal muscle is the primary site of BCAA catabolism, these findings suggest increased utilization of BCAA by skeletal muscle as an energy substrate.

Post-translational modification of amino acids occurs in the liver to enhance AA excretion, and levels of several modified amino acids (by glycosylation, acetylation, and formylation), were enhanced in the severe AAH cohort (**Table 2**). Of note, N-acetyltryptophan levels were increased 421-fold in severe AAH compared to cirrhotic controls, and N-acetyl-serine was identified in the RF analysis as a key component of the metabolic signature of AAH. There was a trend towards increased urea levels with decreased arginine and citrulline levels in severe AAH (**Figure S7C**), reflecting increased hepatic disposal of nitrogenous compounds, including amino acids. Finally, several dipeptides (phenylalanyltryptophan, leucylalanine, prolylhydroxyproline, phenylalanylphenylalanine, and phenylalanylleucine) were detected in the RF analysis discriminating severe AAH from alcoholic cirrhosis (**Figure 1** **,** **Table 2**). Together, these findings highlight the importance of increased protein degradation and amino acid catabolism for maintaining energy homeostasis in severe AAH.

**Table 2:** Serum levels of protein degradation products.

Metabolite	Fold Change (AAH vs. Cirrhosis)	*P* Value	*Q* Value
**N-acetylglycine**	1.74	<0.001	<0.001
**N-acetylserine**	2.66	<0.001	<0.001
**N-acetylthreonine**	1.99	0.0038	0.0033
**N-acetyl-beta-alanine**	1.49	<0.001	<0.001
**N-acetylalanine**	1.78	<0.001	<0.001
**N6-acetyllysine**	1.44	<0.001	<0.001
**N-acetylphenylalanine**	1.72	0.0033	0.0029
**N-acetyltryptophan**	421.50	0.0102	0.0072
**N-acetylmethionine**	1.62	0.0035	0.003
**Aspartylphenylalanine**	0.48	<0.001	<0.001
**Prolylalanine**	1.51	0.0036	0.0031
**Leucyleucine**	0.64	0.0166	0.0105
**Pro-hydroxyproline**	2.57	<0.001	<0.001
**Phenylalanylphenylalanine**	0.25	<0.001	<0.001
**Valylarginine**	0.43	<0.001	<0.001
**Histidyltryptophan**	0.40	<0.001	<0.001
**Leucylalanine**	0.31	<0.001	<0.001
**Phenylalanylleucine**	0.31	<0.001	<0.001
**Phenylalanylserine**	0.54	<0.001	<0.001
**Phenylalanyltyptophan**	0.24	<0.001	<0.001

Relative levels of serum protein degradation products, including acetylated amino acids, dipeptides, and urea cycle intermediates. Serum levels of each metabolite were compared using paired Welch's *t* tests, and *q* values were calculated to account for false discovery rates to correct for multiple comparisons.

### Metabolic alterations in severe AAH indicate an oxidative environment and increased inflammation

Altered levels of several biomolecules highlighted the contributions of oxidative stress, antioxidant depletion and inflammation to metabolic phenotypes in severe AAH. In addition to enhanced levels of pentose phosphate pathway metabolites, serum symmetric dimethylarginine (SDMA), an important regulator of monocyte endothelial ROI generation [26] was significantly increased in patients with severe AAH versus cirrhotic controls. As SDMA undergoes renal excretion, serum levels may reflect subclinical renal dysfunction in this cohort. Other oxidized metabolites, including biliverdin and erythronate were also greater in AAH (**Figure S8**). Furthermore, three of six detected monohydroxylated fatty acids were increased, as was 1,2-propanediol, a metabolite derived from methylglyoxal detoxification [27]. Together, these findings reflect enhanced oxidative stress in severe AAH.

Conversely, the metabolomics analysis revealed evidence of altered antioxidant utilization in severe AAH. Glutathione is a key antioxidant synthesized both from de novo and salvage pathways within in the liver. In patients with severe AAH, levels of both alpha-ketobutyrate and alpha-hydroxybutyrate were significantly increased, suggesting shunting of homocysteine to glutathione synthesis. Interestingly, this was accompanied by increased methionine levels, which may reflect decreased utilization of methionine for S-adenosyl methionine (SAMe) generation. Finally, there was a trend towards higher levels of 5-oxoproline (*p* = 0.066), a marker of glutathione degradation. In addition, significant reductions in ascorbate (vitamin C) and gamma-CEHC (a product of gamma-tocopherol metabolism) were observed, indicating either antioxidant depletion or malnutrition (**Figure S8**).

**Figure 9:**
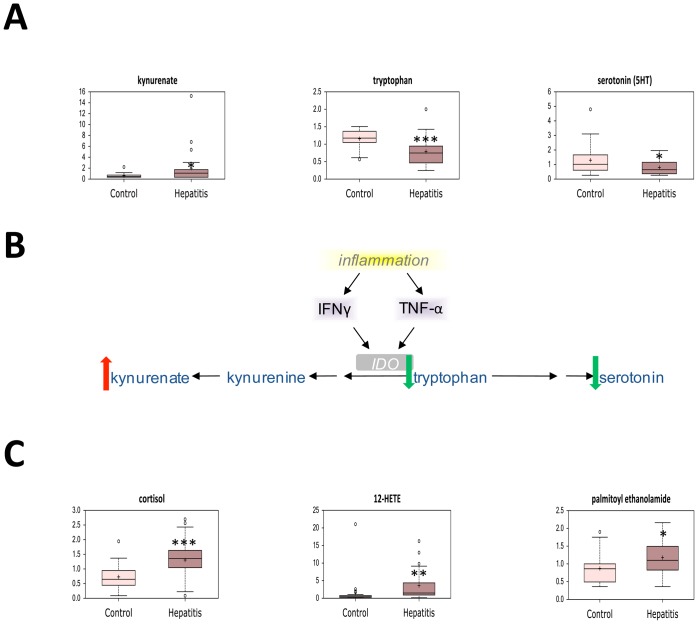
Alterations in inflammation-associated metabolites in AAH. (A) Serum levels of tryptophan metabolites in patients with severe acute alcoholic hepatitis (hepatitis) and stable alcoholic cirrhosis (control). (B) Schematic representation of altered indoleamine 2,3-dioxygenase activity in severe AAH. (C) Serum levels of lipid-derived inflammatory mediators in patients with severe acute alcoholic hepatitis (hepatitis) and stable alcoholic cirrhosis (control). * *p*<0.05, ** *p*<0.01, *** *p*<0.001. IDO, indoleamine 2,3-dioxygenase; TNF-α, tumor necrosis factor α; IFN-γ, interferon-γ.

In conjunction with increased oxidative stress and decreased antioxidant capacity, altered levels of several biochemicals highlighted the profound inflammation seen in acute AAH. Indoleamine 2,3-dioxygenase (IDO) is a highly-inducible enzyme which is up-regulated by the inflammatory cytokines tumor necrosis factor α (TNFα) and interferon-gamma (IFN-γ) [28], [29]. Tryptophan was preferentially shunted towards IDO metabolism and away from serotonin synthesis, as the IDO product kynenurate was increased while serotonin levels were decreased in patients with severe AAH (**Figure 9A,B**). Together, these findings reflect increased TNF-α and IFN-γ-mediated inflammation. In addition, serum levels of cortisol as well as the proinflammatory molecules 12-HETE and palmitoyl ethanolamide were significantly higher in patients with severe AAH (**Figure 9C**). In total, these markers of oxidative stress and inflammation confirm known associations between these pathways and the pathogenesis of AAH.

### A panel of metabolic biomarkers identify long-term survivors with severe AAH

As only 56% of patients with severe AAH survived to 180 days, we sought to identify metabolic biomarkers associated with mortality. Treatments likely did not explain differences in survival of patients with severe AAH, as similar proportions of survivors and non-survivors received prednisolone (31.4% of survivors versus 42.6% of non- survivors, *p* = N.S.), pentoxifylline (64.5% versus 70.9%, *p* = N.S.), both medications (5.7% versus 11.6%, *p* = N.S.) or neither medication. (4.7% versus 9.8%, *p* = N.S.). Overall, levels of twenty named metabolites were significantly different between survivors and non- survivors with severe AAH (**Table 3**). Of these, four biochemicals were elevated in survivors, while 16 were decreased. Multiple metabolites that were altered between severe AAH and alcoholic cirrhosis also differed between survivors and nonsurvivors. In particular, the biomolecule 3-hydroxybutyrate, which was identified in the RF analysis distinguishing severe AAH from alcoholic cirrhosis, was also elevated in survivors of severe AAH.

**Table 3:** Serum levels of significantly altered metabolites between AAH survivors and nonsurvivors.

Pathway	Metabolite	Fold Change*	*P*	*Q*
**Glutamate Metabolism**	**glutamine**	0.72	0.0285	0.4415
**Aromatic AA Metabolism**	**3-methoxytyrosine**	1.39	0.042	0.4415
**BCAA Metabolism**	**3-hydroxyisobutyrate**	1.72	0.046	0.4415
**SH-Containing AA Metabolism**	**α-ketobutyrate**	2.76	0.044	0.4415
	**2-hydroxybutyrate**	1.82	0.04523	0.4415
	**S-methylcysteine**	0.75	0.0226	0.4415
**Dipeptide Metabolism**	**valylarginine**	0.45	0.0215	0.4415
**Glucose Metabolism**	**glucose**	1.22	0.0454	0.4415
**Pentose Phosphate Pathway**	**threitol**	2.04	0.0499	0.4415
**Mannose Metabolism**	**mannose**	1.46	0.0358	0.4415
**Branched FA Metabolism**	**17-methylstearate**	1.49	0.0398	0.4415
**Acyl Carnitine Metabolism**	**hexanoylcarinitine**	1.72	0.0433	0.4415
	**decanoylcarnitine**	1.60	0.0336	0.4415
**Lysolipid Metabolism**	**1-linoleoylglycerophosphoethanolamine**	0.39	0.0182	0.4415
**Sterol Metabolism**	**β-sitosterol**	1.79	0.0430	0.4415
**Steroid Metabolism**	**21-hydroxypregnenolone disulfate**	2.83	0.0049	0.4415
	**pregn-steroid monosulfate**	2.88	0.0309	0.4415
	**4-androsten-3-beta,17-betadiol disulfate**	1.89	0.0427	0.4415
**Purine Metabolism**	**N1-methyladenosine**	1.20	0.0429	0.4415
**Porphyrin Metabolism**	**heme**	1.88	0.0280	0.4415

Relative levels of serum metabolites between survivors and nonsurvivors with severe AAH. Serum levels of each metabolite were compared using paired Welch's *t* tests, and *q* values were calculated to account for false discovery rates to correct for multiple comparisons. AA, amino acid; BCAA, branched-chain amino acid; FA, fatty acid. *Fold change Survivor vs. Non-survivor.

Next, we performed univariable exact logistic regression analysis to determine predictors of 6-month survival in severe AAH. Of the twenty biomarkers studied, 15 were significantly associated with mortality in this cohort (**Table 4**).

**Table 4:** Metabolic predictors of 180-day survival in severe AAH by logistic regression analysis.

Metabolite	Univariate O.R.	95% C.I.	*P*
**glutamine**	23.994	1.215–1192.400	0.034
**3-methoxytyrosine**	0.084	0.005–0.825	0.032
**3-hydroxyisobutyrate**	0.400	0.114–0.987	0.045
**α-ketobutyrate**	0.339	0.095–0.862	0.018
**2-hydroxybutyrate**	0.510	0.229–0.968	0.038
**S-methylcysteine**	31.712	1.334–2131.335	0.028
**valylarginine**	30.848	1.705–1669.992	0.013
**glucose**	0.917	0.485–1.718	0.778
**threitol**	0.743	0.453–1.067	0.124
**mannose**	0.128	0.013–0.746	0.018
**17-methylstearate**	0.196	0.028–0.918	0.037
**hexanoylcarinitine**	0.530	0.218–1.037	0.066
**decanoylcarnitine**	0.297	0.047–1.006	0.052
**1-linoleoylglycerophosphoethanolamine**	15.767	1.129–737. 559	0.033
**β-sitosterol**	0.228	0.285–0.987	0.047
**21-hydroxypregnenolone disulfate**	0.206	0.028–0.802	0.009
**pregn-steroid monosulfate**	0.262	0.039–0.900	0.025
**4-androsten-3-beta,17-betadiol disulfate**	0.706	0.459–0.993	0.045
**N1-methyladenosine**	0.035	0.001–0.985	0.049
**heme**	0.680	0.349–1.148	0.174

Univariable logistic regression analysis for metabolic predictors of 180-day survival in severe AAH. AA, amino acid; BCAA, branched-chain amino acid; FA, fatty acid; O.R., odds ratio; C.I., confidence interval.

## Discussion

In this prospective case control study, we utilized unbiased serum metabolomics to characterize alterations in pathways associated with severe AAH. Three important observations were noted. First, we demonstrated that changes in serum metabolites accurately discriminated AAH from alcoholic cirrhosis. Next, we described differences in biochemical levels related to several metabolic pathways, including lipid metabolism, bile acid homeostasis, protein utilization, glucose disposal, oxidative stress, and inflammation. Finally, we identified a panel of metabolic biomarkers that differed between survivors and nonsurvivors with severe AAH for future analysis.

Random forest (RF) classification of serum metabolic profiles distinguished severe AAH from alcoholic cirrhosis with 100% accuracy, thus indicating that differences between the two groups were quite pronounced. RF analysis was also used to identify the 30 most important biochemicals separating severe AAH from alcoholic cirrhosis, and principal component analysis (PCA) demonstrated that metabolic signatures of severe AAH varied greatly from those related to alcoholic cirrhosis. Together, RF analysis and PCA provide compelling evidence that severe AAH is characterized by a distinct metabolic phenotype spanning several metabolic pathways.

Animal models of alcoholic liver disease have demonstrated dysfunctional adipose and hepatic lipid metabolism, including adipose tissue hyperlipolysis, defective hepatic lipid catabolism, and altered lipid trafficking [7]–[9], [12], [30]. Similar to these findings, patients with severe AAH in the current study exhibited enhanced adipose tissue triglyceride lipolysis, relative impairment of hepatic fatty acid beta oxidation, and enhanced fatty acid omega oxidation. In particular, accumulation of incomplete LCFA degradation products suggests reduced mitochondrial oxidative capacity for a large FA load. In addition to enhanced adipose tissue lipolysis and dysfunctional fatty acid oxidation, changes in other lipid-derived metabolites were also observed. Low serum levels of multiple phospholipids, lysolipids, and associated degradation products suggest decreased cell membrane remodeling in severe AAH [31], [32].

While levels of most serum bile acids were elevated in patients with severe AAH, levels of two gut microbiota-derived moieties, deoxycholate and glycodeoxycholate, were decreased. Additionally, pronounced changes in enteric microbial benzoate handling were identified in patients with severe AAH. Similar phenomena have been described in metabolomics studies of intestinal microbial alteration in inflammatory conditions such as obesity and inflammatory bowel disease. Together, these findings highlight a role for intestinal dysbiosis in severe AAH [33]–[35].

Metabolomics profiling also highlighted several changes in substrate utilization for energy homeostasis. The accumulation of several sugar alcohols in patients with severe AAH indicated glucose shunting towards the pentose phosphate pathway, an important source of reduced nicotinamide adenine dinucleotide (NADH) to neutralize oxidative stress. Observed elevations in serum lactate levels may be related to impaired hepatic clearance, as previous human and animal studies have demonstrated decreased gluconeogenesis in the setting of alcohol consumption [36]. Increased levels of multiple TCA cycle intermediates in patients with severe AAH reflect decreased mitochondrial energy metabolism, and high levels of multiple protein degradation products pointed to increased utilization of amino acids as a primary energy substrate in this population.

The metabolic phenotype of AAH is characterized by oxidative stress resulting in systemic inflammation [37], [38], and to this effect levels of several oxidized metabolites were elevated in severe AAH. Pronounced depletion of antioxidants, including vitamin C and the vitamin A metabolite gamma-CEHC was observed, and multiple metabolites related to glutathione turnover were altered. These changes were associated with increased kynenurate, decreased serotonin, and decreased tryptophan levels, likely mediated by the highly-inducible enzyme IDO. As IDO expression is up-regulated by TNFα and IFN-γ [28], [29], these findings underscore the role of systemic inflammation in severe AAH.

Finally, we compared metabolite levels in survivors and non-survivors with severe AAH to determine potential biomarkers of disease severity and prognosis. Twenty metabolites spanning multiple metabolic pathways were significantly different between groups, and of these biomarkers, 15 were significantly associated with 6-month survival by univariable logistic regression analysis. This difference was not related to treatment effects, as exposure to corticosteroids and pentoxifylline was similar between survivors and non-survivors. Together, these metabolites represent a potential biomarker panel for disease prognosis and may shed light on the pathogenesis of severe AAH.

A few limitations were noted. First, the serum metabolome represents the net result of metabolic changes in several tissues; nonetheless, many of the biomolecules identified in the current study were primarily derived from energy homeostasis organs, including the liver, adipose tissue, and skeletal muscle. Second, quantification of ethanol consumption was not obtained from most patients, and many of the metabolic pathways studied are directly regulated by ethanol. Even so, levels of ethyl glucuronide, a derivative of ethanol metabolism commonly used to assess long-term abstinence [39], [40], were similar between patients with severe AAH and alcoholic cirrhosis, and all patients with severe AAH underwent blood collection within 24 hours of admission. Finally, small sample sizes (n = 25 per group) may have increased the probability of type II error in hypothesis testing. Despite the sample size, however, a distinct metabolic phenotype for severe AAH was defined.

In conclusion, our findings define the rich metabolic signature of severe AAH and identify potential metabolic biomarkers of disease prognosis. Future studies are planned to validate these findings in larger cohorts of patients with severe AAH.

## Supporting Information Legends

Figure S1 **Unbiased hierarchical clustering analysis of measured metabolites between subject cohorts.**
(TIF)

Figure S2 **Serum levels of long chain free fatty acids (LCFA) and triglyceride hydrolysis intermediates in patients with severe acute alcoholic hepatitis (hepatitis) and stable alcoholic cirrhosis (control).** (A) Long chain free fatty acids. (B) Triglyceride hydrolysis intermediates. * *p*<0.05, ** *p*<0.01, *** *p*<0.001.
(TIF)

Figure S3 **Serum levels of short-(SCFA) and medium-chain fatty acids (MCFA), fatty acylcarnitines, and ketone bodies in patients with severe acute alcoholic hepatitis (hepatitis) and stable alcoholic cirrhosis (control).** (A) Short- and medium-chain fatty acids. (B) Fatty acylcarnitines. (C) Ketone bodies. * *p*<0.05, ** *p*<0.01, *** *p*<0.001.
(TIF)

Figure S4 **Serum levels of dicarboxylic acids in patients with severe acute alcoholic hepatitis (hepatitis) and stable alcoholic cirrhosis (control).** *p*<0.05, ** *p*<0.01, *** *p*<0.001.
(TIF)

Figure S5 **Serum levels of lysolipids and intermediates of lysolipid and phospholipid metabolism in patients with severe acute alcoholic hepatitis (hepatitis) and stable alcoholic cirrhosis (control).** (A) Lysolipids. (B) Intermediates of lysolipid and phospholipid metabolism. * *p*<0.05, ** *p*<0.01, *** *p*<0.001.
(TIF)

Figure S6 **Serum levels of glucose utilization pathway and tricarboxylic acid (TCA) cycle intermediates in patients with severe acute alcoholic hepatitis (hepatitis) and stable alcoholic cirrhosis (control).** (A) Glucose with associated metabolic products. (B) Pentose phosphate pathway intermediates. (C) Glucuronate and 2,3-butanediol. (D) Tricarboxylic acid (TCA) cycle intermediates. * *p*<0.05, ** *p*<0.01, *** *p*<0.001.
(TIF)

Figure S7 **Serum levels of branched-chain amino acids (BCAA), BCAA degradation products, and urea cycle intermediates in patients with severe acute alcoholic hepatitis (hepatitis) and stable alcoholic cirrhosis (control).** (A) Branched-chain amino acids. (B) BCAA degradation products. (C) Urea cycle intermediates. * *p*<0.05, ** *p*<0.01, *** *p*<0.001.
(TIF)

Figure S8 **Serum levels of oxidized biomolecules, monohydroxy fatty acids, intermediates of glutathione metabolism and antioxidants in patients with severe acute alcoholic hepatitis (hepatitis) and stable alcoholic cirrhosis (control).** (A) Oxidized biomolecules. (B) Monohydroxy fatty acids. (C) Intermediates of glutathione metabolism. (D) Antioxidants. * *p*<0.05, ** *p*<0.01, *** *p*<0.001.
(TIF)

Table S1 **Serum metabolite concentrations from patients with severe acute alcoholic hepatitis (hepatitis) and stable alcoholic cirrhosis (control).**
(XLSX)
