# Supplementary figures and images for: Serum Metabolomic Profiling in Acute Alcoholic Hepatitis Identifies Multiple Dysregulated Pathways
Source: PLoS One. 2014 Dec 2;9(12):e113860. doi: 10.1371/journal.pone.0113860 (PMC4252257; doi:10.1371/journal.pone.0113860)

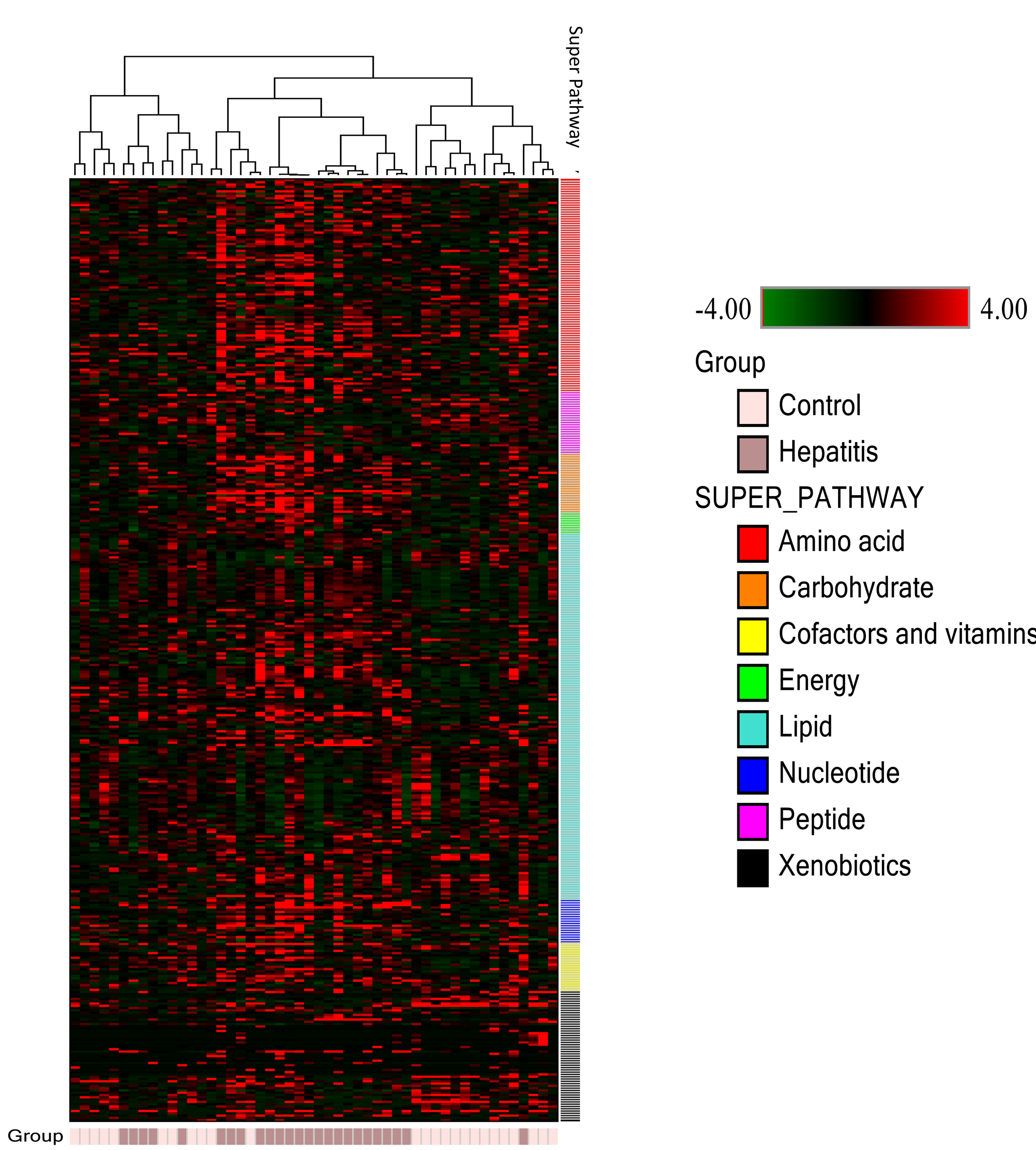

Supplement: Figure S1 — Unbiased hierarchical clustering analysis of measured metabolites between subject cohorts. (TIF) [file pone.0113860.s001.tif]

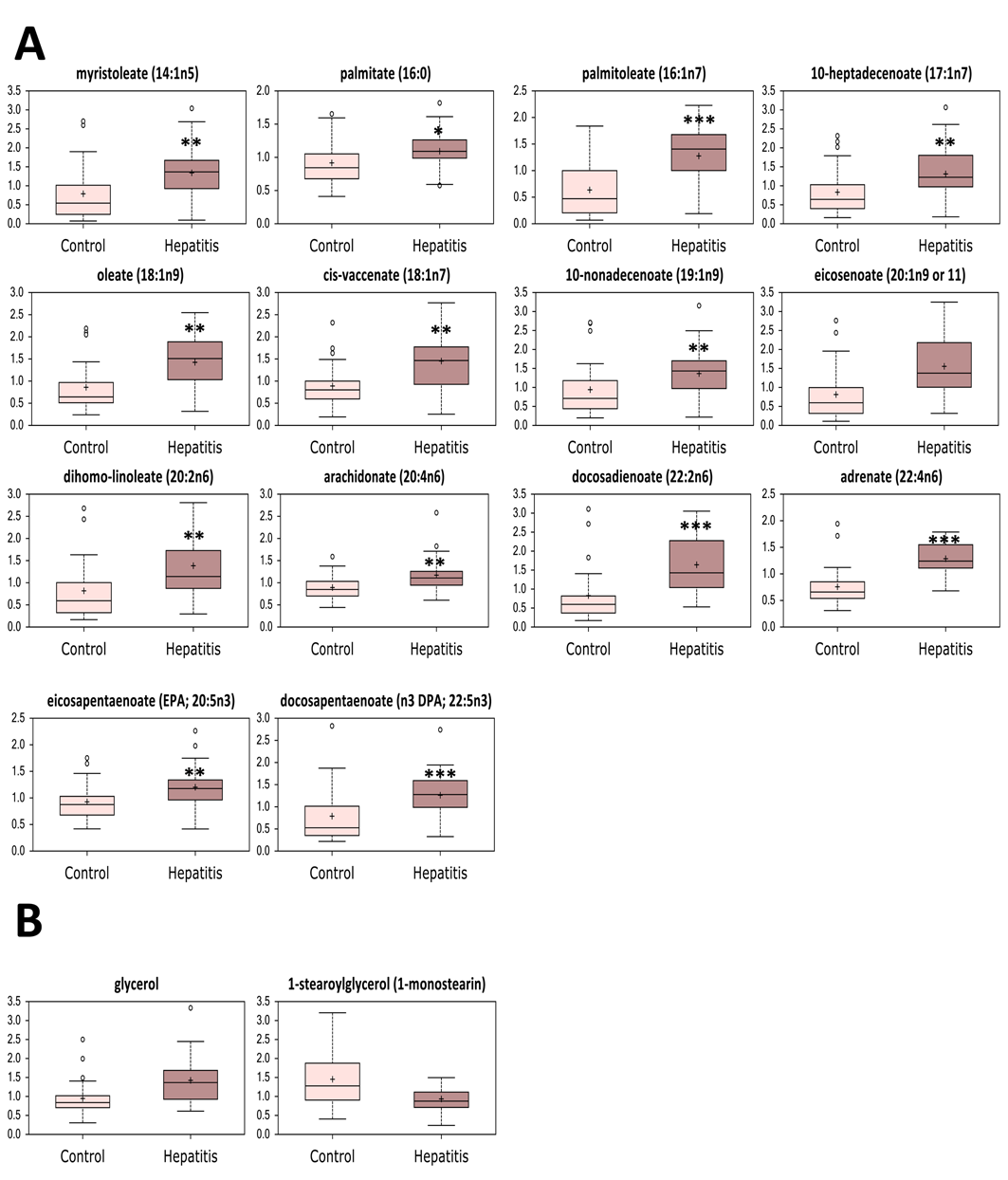

Supplement: Figure S2 — Serum levels of long chain free fatty acids (LCFA) and triglyceride hydrolysis intermediates in patients with severe acute alcoholic hepatitis (hepatitis) and stable alcoholic cirrhosis (control). (A) Long chain free fatty acids. (B) Triglyceride hydrolysis intermediates. * p<0.05, ** p<0.01, *** p<0.001. (TIF) [file pone.0113860.s002.tif]

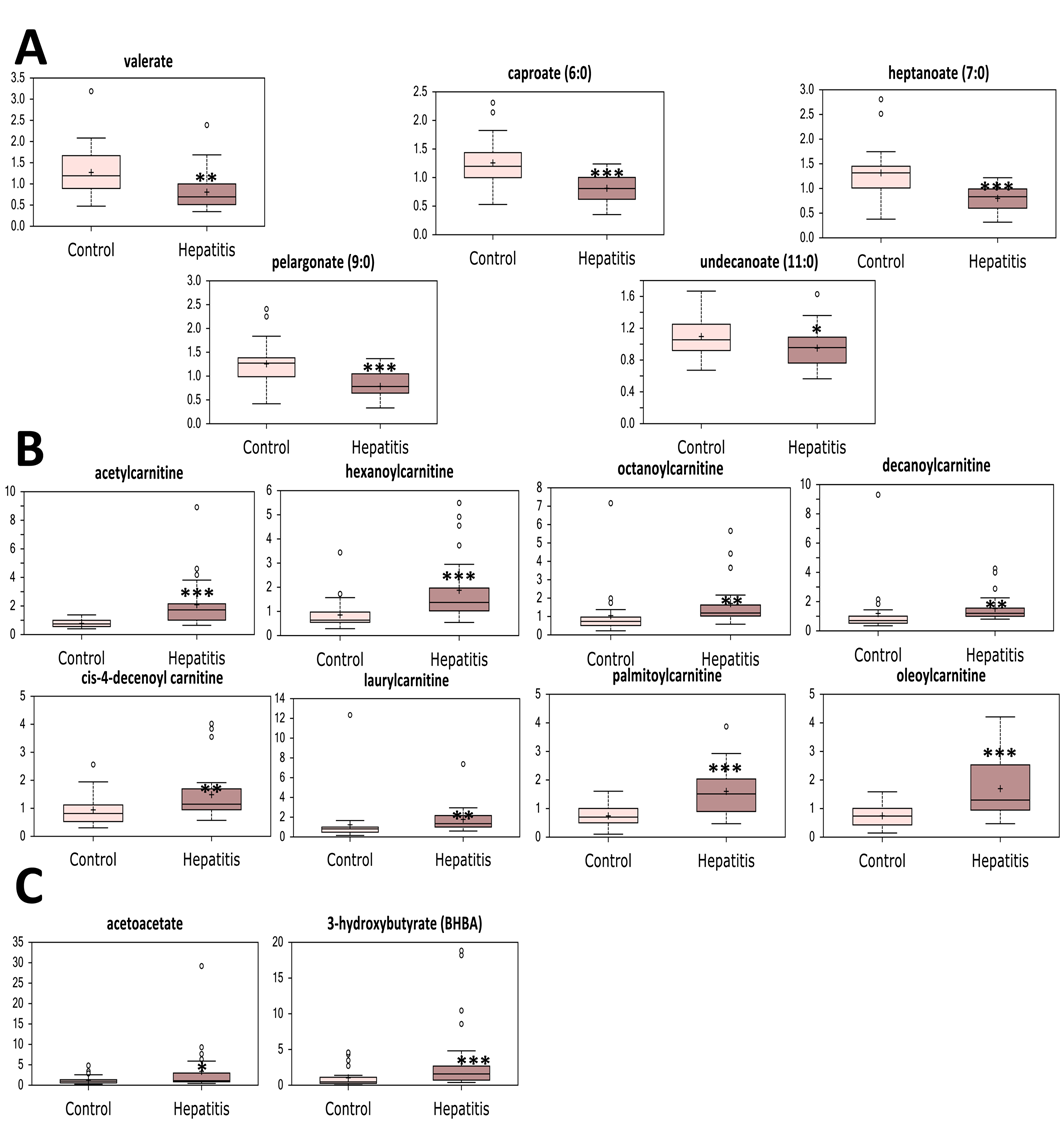

Supplement: Figure S3 — Serum levels of short-(SCFA) and medium-chain fatty acids (MCFA), fatty acylcarnitines, and ketone bodies in patients with severe acute alcoholic hepatitis (hepatitis) and stable alcoholic cirrhosis (control). (A) Short- and medium-chain fatty acids. (B) Fatty acylcarnitines. (C) Ketone bodies. * p<0.05, ** p<0.01, *** p<0.001. (TIF) [file pone.0113860.s003.tif]

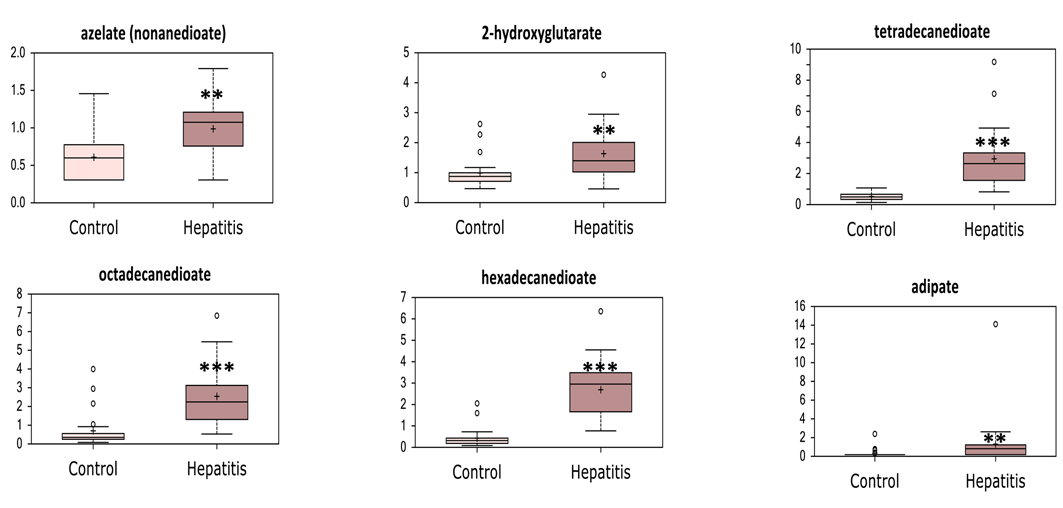

Supplement: Figure S4 — Serum levels of dicarboxylic acids in patients with severe acute alcoholic hepatitis (hepatitis) and stable alcoholic cirrhosis (control). p<0.05, ** p<0.01, *** p<0.001. (TIF) [file pone.0113860.s004.tif]

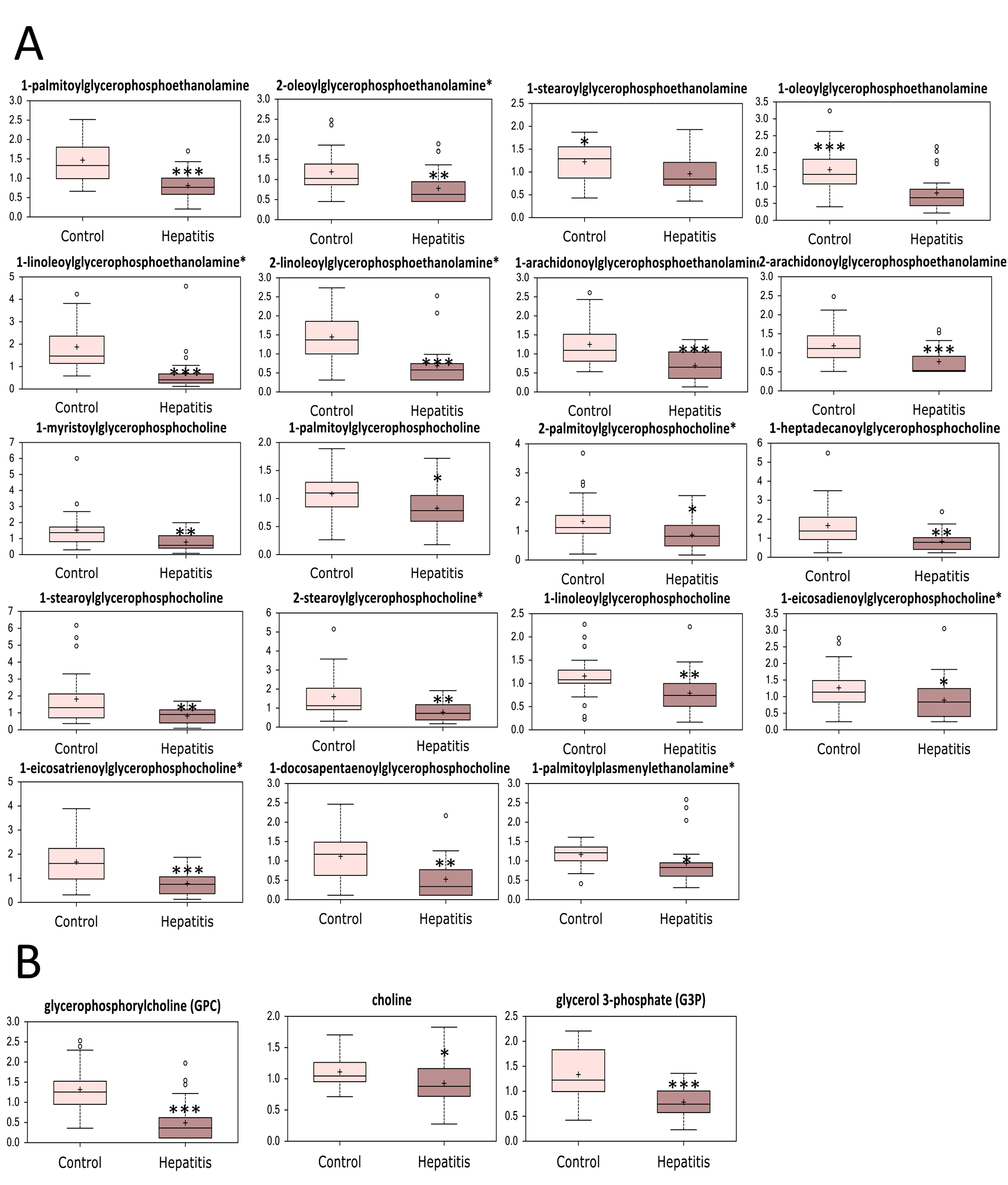

Supplement: Figure S5 — Serum levels of lysolipids and intermediates of lysolipid and phospholipid metabolism in patients with severe acute alcoholic hepatitis (hepatitis) and stable alcoholic cirrhosis (control). (A) Lysolipids. (B) Intermediates of lysolipid and phospholipid metabolism. * p<0.05, ** p<0.01, *** p<0.001. (TIF) [file pone.0113860.s005.tif]

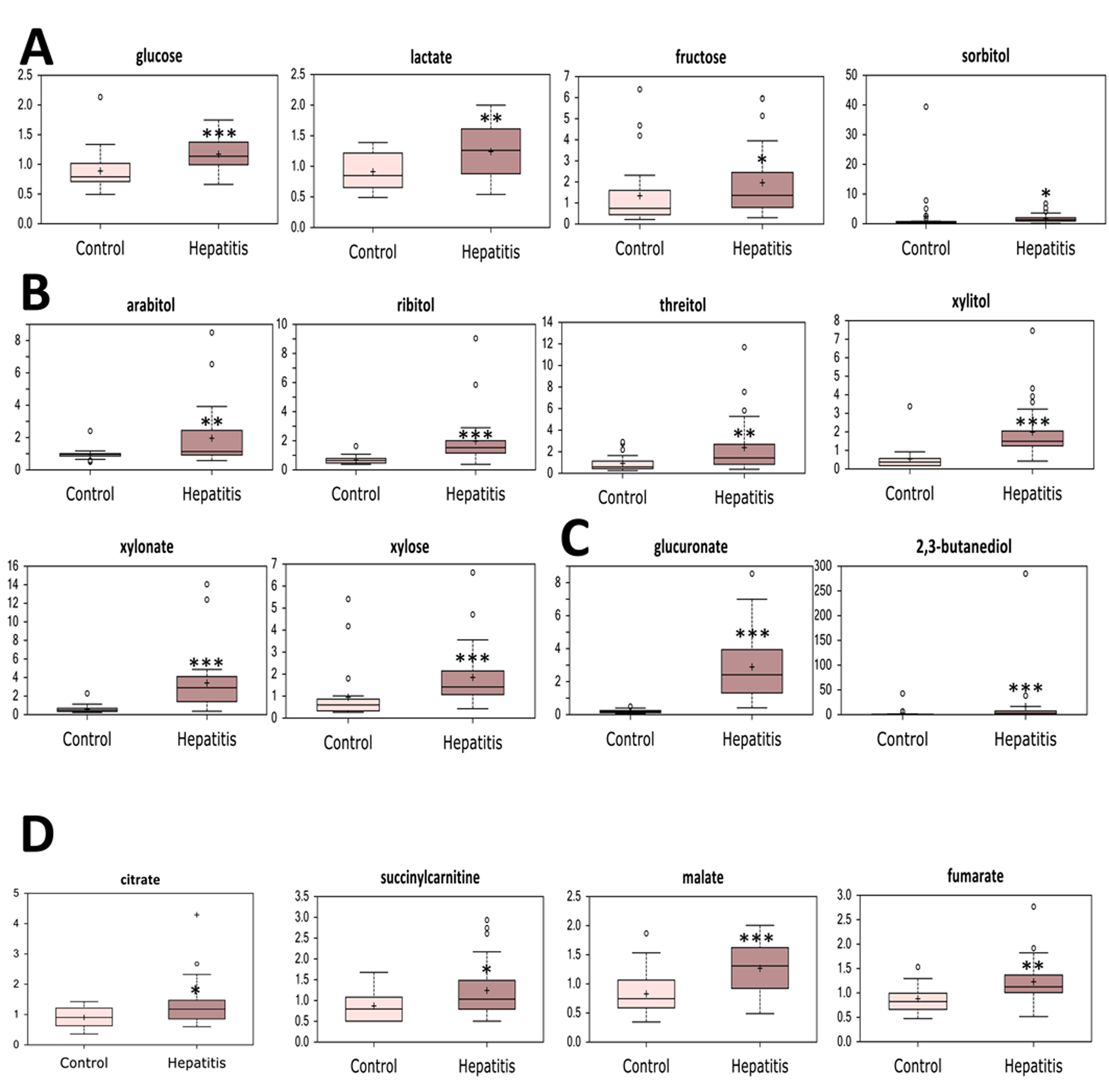

Supplement: Figure S6 — Serum levels of glucose utilization pathway and tricarboxylic acid (TCA) cycle intermediates in patients with severe acute alcoholic hepatitis (hepatitis) and stable alcoholic cirrhosis (control). (A) Glucose with associated metabolic products. (B) Pentose phosphate pathway intermediates. (C) Glucuronate and 2,3-butanediol. (D) Tricarboxylic acid (TCA) cycle intermediates. * p<0.05, ** p<0.01, *** p<0.001. (TIF) [file pone.0113860.s006.tif]

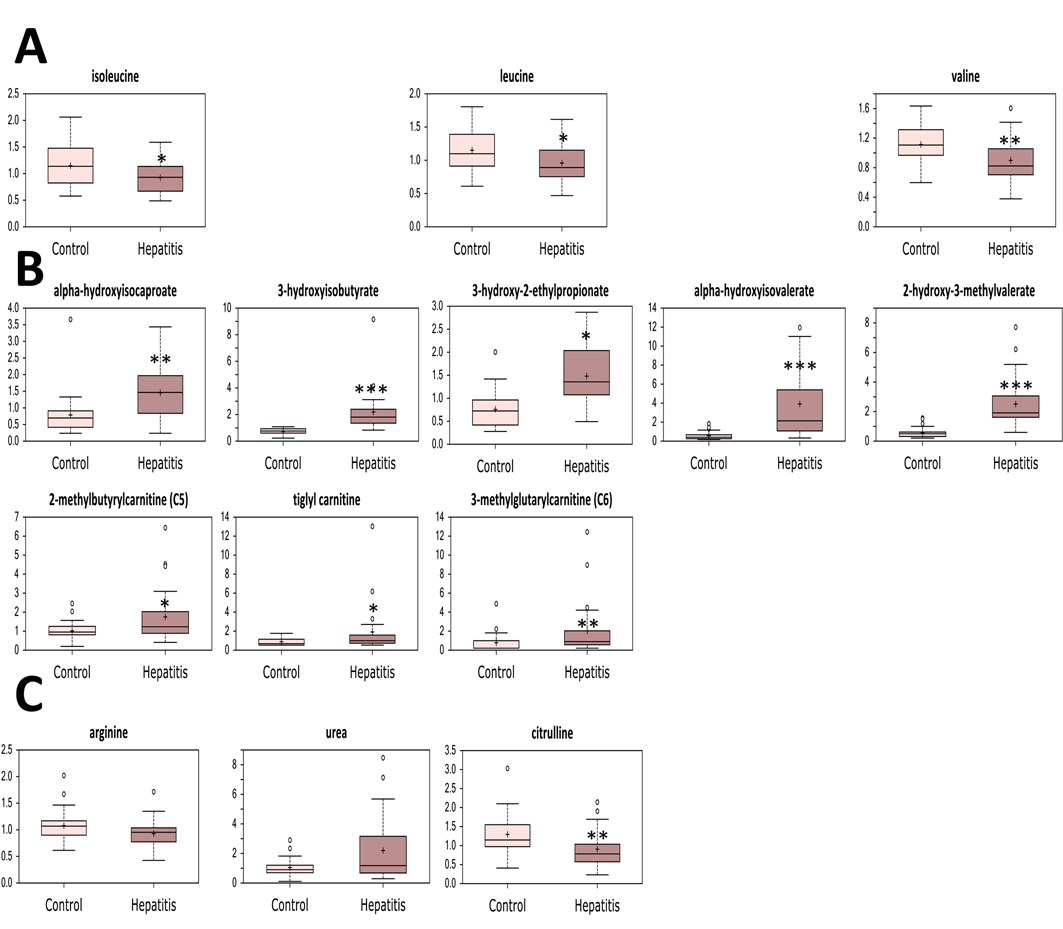

Supplement: Figure S7 — Serum levels of branched-chain amino acids (BCAA), BCAA degradation products, and urea cycle intermediates in patients with severe acute alcoholic hepatitis (hepatitis) and stable alcoholic cirrhosis (control). (A) Branched-chain amino acids. (B) BCAA degradation products. (C) Urea cycle intermediates. * p<0.05, ** p<0.01, *** p<0.001. (TIF) [file pone.0113860.s007.tif]

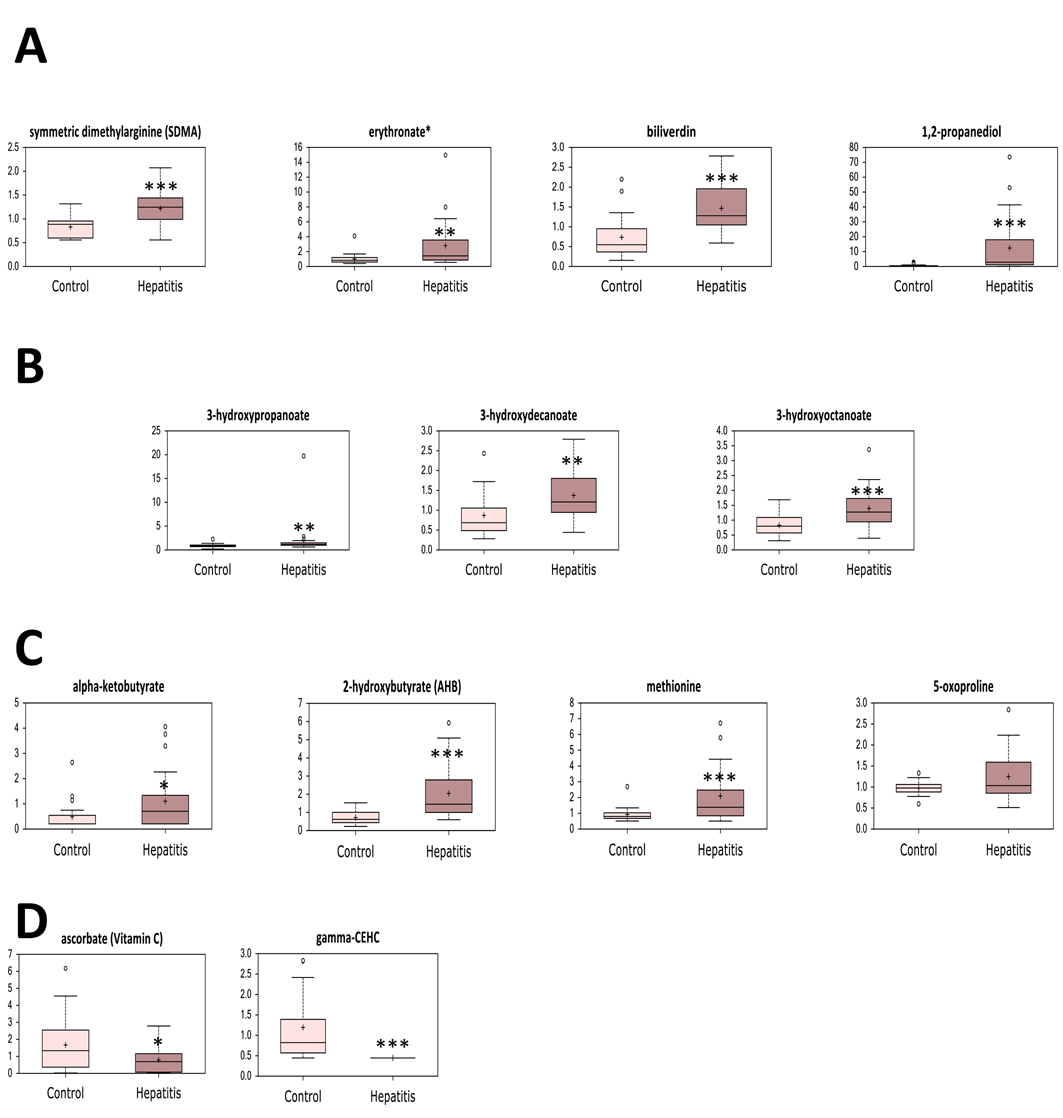

Supplement: Figure S8 — Serum levels of oxidized biomolecules, monohydroxy fatty acids, intermediates of glutathione metabolism and antioxidants in patients with severe acute alcoholic hepatitis (hepatitis) and stable alcoholic cirrhosis (control). (A) Oxidized biomolecules. (B) Monohydroxy fatty acids. (C) Intermediates of glutathione metabolism. (D) Antioxidants. * p<0.05, ** p<0.01, *** p<0.001. (TIF) [file pone.0113860.s008.tif]
